# Self-Reported Pre-Pandemic Physical Activity and Likelihood of COVID-19 Infection: Data from the First Wave of the CoCo-Fakt Survey

**DOI:** 10.1186/s40798-023-00592-6

**Published:** 2023-06-21

**Authors:** Nikola Schmidt, Andreas Gehlhar, Barbara Grüne, Annelene Kossow, Thomas Kraus, Johannes Nießen, Stefanie Wessely, Christine Joisten

**Affiliations:** 1 Department for Physical Activity in Public Health, Institute of Movement and Neurosciences, German Sport University Cologne, Cologne, Germany; 2 Department of Infection Control and Environmental Hygiene, Cologne Health Authority, Cologne, Germany; 3 Institute of Hygiene, University Hospital Münster, Münster, Germany; 4 Institute for Occupational, Social and Environmental Medicine, Uniklinik RWTH Aachen University, Aachen, Germany; 5 Cologne Health Authority, Cologne, Germany

**Keywords:** SARS-CoV-2, COVID-19 infection, Exercise, Physical activity, Risk factors

## Abstract

**Objectives:**

To investigate the potential protective role of exercise on the odds of COVID-19 infection in unvaccinated contact persons (CPs) who were at higher risk of infection due to confirmed contact with infected persons.

**Methods:**

Before the onset of the vaccination campaign, the first wave of the CoCo-Fakt online survey was conducted with SARS-CoV-2-positive persons and their confirmed contacts who were isolated/quarantined between 1 March 2020 and 9 December 2020. Within this analysis, 5338 CPs were included and divided into those who subsequently tested positive (CP-P) and those who remained negative (CP-N). We assessed demographics as well as pre-pandemic lifestyle characteristics including physical activity (PA; type, frequency, time, intensity; duration clustered as ‘below PA guidelines’, ‘meeting PA guidelines’, and ‘above PA guidelines’; intensity clustered as ‘low intensity’ and ‘moderate-to-vigorous intensity’) and sedentary behaviour.

**Results:**

A greater percentage of CP-Ns reported being active before the pandemic compared to CP-Ps (69% vs. 63%; *p* = .004). Moreover, CP-Ns reported higher PA duration (164.1 min/week vs. 143.2 min/week; *p* = .038) and higher PA intensities than CP-Ps (67% vs. 60% moderate-to-vigorous intensity, 33% vs. 40% low intensity; *p* = .003). Adjusting for age, sex, socioeconomic status, migration background, and pre-existing chronic diseases, the odds of infection were negatively associated with exercise (yes/no) (Nagelkerke R^2^ = 1.9%), PA levels above PA guidelines (Nagelkerke R^2^ = 2.0%), and PA intensity (Nagelkerke R^2^ = 1.8%).

**Conclusion:**

Due to the beneficial effects of PA on the odds of infection, an active lifestyle should be promoted especially during possible subsequent pandemics (while taking into account necessary hygiene measures). Moreover, inactive and chronically ill persons should be especially encouraged to adopt a healthier lifestyle.

**Supplementary Information:**

The online version contains supplementary material available at 10.1186/s40798-023-00592-6.

## Key Points

- In unvaccinated persons who had confirmed contact with a COVID 19-infected person, pre-pandemic physical activity was associated with lower odds of infection.
- Engaging in physical activity at all, but also higher intensities and durations resulted in reduced odds of infection.

## Introduction

Although, 3 years on, the coronavirus disease 2019 (COVID-19) pandemic is gradually turning into an endemic-wave occurrence [1], it remains an incisive episode that leaves unanswered questions, also with regard to possible future pandemics. Before the onset of the vaccination campaign, elderly patients and patients with chronic diseases were at especially high risk of severe courses and death [2, 3], which led to strict measures such as social distancing, lockdowns, and mask-wearing [4]. Though these measures also entailed negative consequences such as psychological perturbations [5], poorer sleep patterns [6], and changes in lifestyle [6–8], they were, along with vaccination, the most important factors in avoiding the overloading of the health care system and fatal courses [4]. However, considering the substantial psychological stress caused by such rigorous measures, further possibilities for the prevention of COVID-19 should be investigated. In the few studies available to date (March 2023), physical activity (PA) before a COVID-19 infection has been shown to have a beneficial effect on infectivity and outcomes, such as hospitalisation, intensive care unit admission, mortality, and severity and number of post-COVID-19 symptoms [9–13]. These findings are assumed to be due to the immunomodulatory and anti-inflammatory effects of exercise leading to protection from chronic diseases, reduced risk of severe disease progression, and lower hospitalisation rates in COVID-19 patients [9, 12].

However, most studies have evaluated the infection risk in the general population and not among specifically exposed persons who therefore are at an increased risk of SARS-CoV-2 infection [14, 15]. A contact person (CP) is defined as any person who has had close exposure (< 1.5 m) to a confirmed COVID-19 case for longer than 10 min without adequate protection (FFP2 or medicine mask) and/or any person who has had face-to-face contact (< 1.5 m) without adequate protection, regardless of duration of conversation or direct contact (with respiratory secretions), and/or any person who was present in the same room with a case and probably high concentration of infectious aerosols regardless of distance for longer than 10 min, even if a FFP2 mask was worn continuously and correctly [16]. Therefore, the aim of this analysis was to determine, based on data from the first wave of the CoCo-Fakt cohort study, to what extent PA can protect confirmed contacts without vaccination against infection. Additionally, we investigated whether increased sedentary behaviour increases the odds of getting infected with COVID-19 in CPs.

## Methods

### Study Design

Since the beginning of the SARS-CoV-2 outbreak at the end of February 2020, persons with residence in Cologne who tested positive for SARS-CoV-2 by PCR testing (infected persons, or IPs) were reported to the Cologne Health Authority as required by the German Infection Protection Act. Those persons were contacted, registered using the software “*Digitales Kontakt Management”* [digital contact management] (DiKoMa) [17], quarantined based on the German Infection Protection Act, and interviewed in a standardised manner about possible transmission routes, medical history, symptoms, and relevant CPs. To detect possible infection early and prevent further spread, the CPs were also contacted and quarantined. During the survey phase, isolation or quarantine usually lasted 10 days from symptom onset (for IPs) or 14 days from the last contact (for CPs).

Based on this procedure, the *Cologne-Corona-Beratung und Unterstützung Für Index- und KontAkt-Personen während der Quarantäne-ZeiT* (CoCo-Fakt) survey was conducted. This analysis is part of the CoCo-Fakt cohort study that assessed demographic data, transmission routes, living situation, adherence, psychosocial consequences, coping strategies, and lifestyle during quarantine in three waves. This analysis includes data of the first wave, which was conducted before the vaccination campaign with IPs and their CPs who were isolated/quarantined between 1 March 2020 and 9 December 2020 and utilised the online survey tool Unipark [18]. The questionnaire (Additional file 1) was developed and modified according to the COVID-19 Snapshot Monitoring (COSMO) study [19]. Answering the questionnaire took approximately 30 min, with all questions answered on a voluntary basis. The study protocol was published previously [20].

### Sample

A total of 36,498 persons registered in DiKoMa between February and 9 December 2020 were extracted from the dataset. Exclusion criteria were: under 16 years of age, missing informed consent, noncompliance, deceased patients, and patients in medical or nursing facilities.

**Fig. 1:**
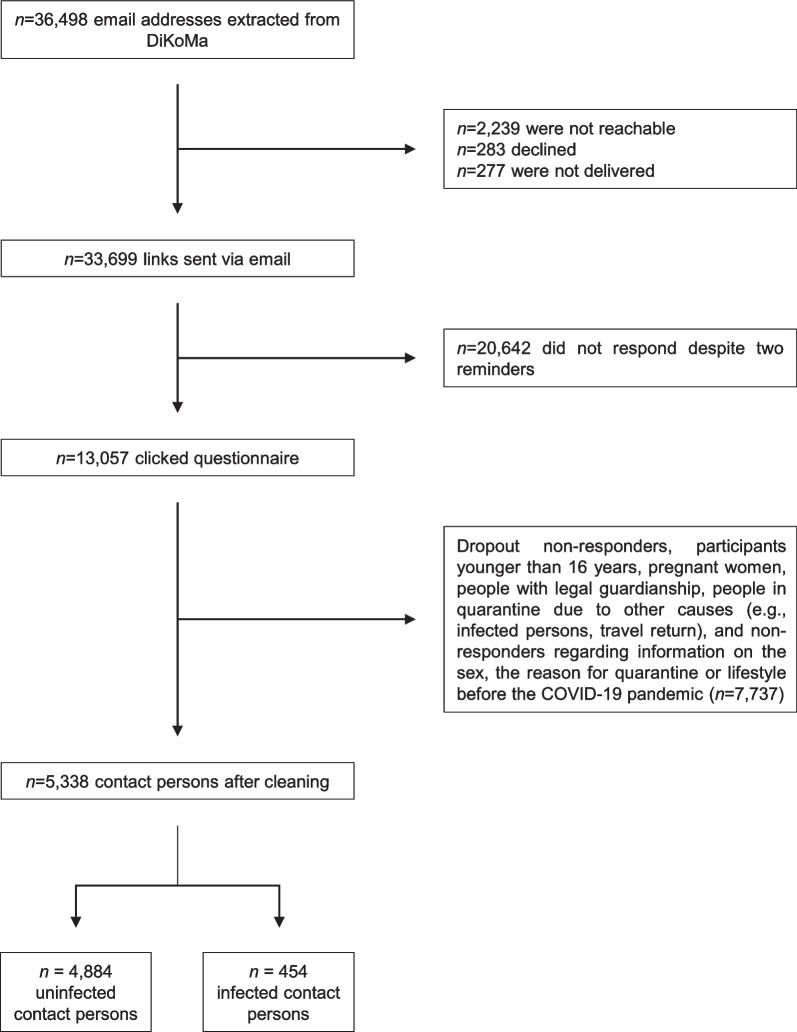
Study population flowchart

An email with information about the background of the study, the summarized content, and an invitation to follow the link to the online survey was sent to 33,699 people, of whom 13,057 gave their informed consent and answered the questionnaire. After cleaning the sample, the CoCo-Fakt cohort comprised 10,547 data records. Of these, the 5338 CPs who provided any information on pre-pandemic PA behaviour were included in this analysis and clustered into groups: CPs who did not test positive for coronavirus (uninfected CP, or CP-N; ‘I was a contact person’, ‘I was a contact person several times’; *n* = 4884) and CPs who subsequently tested positive for coronavirus (infected contact person, or CP-P; ‘I was a contact person and tested positive for the coronavirus afterwards’; *n* = 454) (Fig. 1).

### Survey

The following parameters were assessed:

#### Demographic Data

We assessed age (years), sex (male/female), socioeconomic status (SES), migration background, and smoking status [21]. SES was calculated using the participants’ answers on education and vocational training and categorised as high, middle, or low based on the German Health Interview and Examination Survey for Adults (DEGS) [22]. Migration background was based on the language primarily spoken at home (German = no; any other language = yes). Additionally, we assessed current smoking status (yes/no).

We also recorded whether the subjects had one or more of the following chronic diseases:

- Asthma
- Chronic bronchitis/chronic obstructive pulmonary disease/pulmonary emphysema
- Heart attack/coronary heart disease
- Congestive heart failure
- Stroke
- Diseases of the musculoskeletal system
- Diabetes type 1/type 2
- Hypertension/high blood pressure
- Hypercholesterolemia
- Allergies
- Chronic liver diseases
- Chronic kidney problems/kidney failure
- Depression
- Cancer
- Inflammatory bowel disease
- Other

#### Lifestyle

PA, relaxation, and sedentary behaviour before the pandemic and during the quarantine period were recorded (modified according to [23]). Our analysis only included lifestyle parameters before the pandemic. Daily life activities, such as walking the dog, gardening, and occupational or home activities were excluded.

##### Physical Activity

We assessed whether the subjects were active (yes/no) as well as the type, intensity (very light, light, moderate, vigorous, or very vigorous), frequency (number of sessions per week), and duration (minutes per session) of activity before the pandemic. Based on the type of activity and its intensity, an average baseline metabolic equivalent (MET) value was derived using the Compendium of Physical Activities by Ainsworth et al. [24]. If subjects reported not being active, the baseline MET value was set to 1.0. PA intensity was categorized based on baseline MET value as ‘sedentary behaviour’ (1.0–1.5 METs), ‘light intensity’ (1.6–2.9 METs), ‘moderate intensity’ (3–5.9 METs) or ‘vigorous intensity’ (≥ 6 METs). For the present analysis, the categories ‘sedentary behaviour’ and ‘light intensity’ were combined into ‘low intensity’, while ‘moderate intensity’ and ‘vigorous intensity’ were combined into ‘moderate-to-vigorous intensity’.

PA duration per week was calculated taking into account all activities with self-reported moderate, vigorous, or very vigorous intensities using the following formula:

PA duration per week = PA minutes per week * PA frequency per week.

If the subject reported being inactive, the PA duration per week was set to 0 min.

Based on the World Health Organization (WHO) Guidelines on Physical Activity and Sedentary Behaviour [25] and Bull et al. [26], PA duration was categorised into three PA levels: ‘below PA guidelines’ (< 150 min/week), ‘meeting PA guidelines’ (150 to < 300 min/week), and ‘above PA guidelines’ (≥ 300 min/week).

##### Sedentary Behaviour

Sedentary behaviour was assessed in minutes per week in occupational (office work with and without screen work, driving a car, other) and private settings (using a phone, watching TV, driving a car, reading/writing, hobbies, other). From this data, total time spent sitting, screen time (office work with screen work, using a phone, watching TV), and transportation time (driving a car in occupational and private settings) were summed.

### Data Cleaning and Analysis

Implausible and duplicate data was deleted. Types of PA were converted from answers to open-ended questions into clustered labelled numerical variables. Missing variables were set to the value “9999” and defined as such. Where applicable, missing data was reproduced using the answers to open-ended questions.

We evaluated frequency (percentage), central tendency (mean values) and variation (standard deviation) to describe the sample. Associations between participant characteristics (age, sex, migration background, SES, chronic diseases, smoking status) and lifestyle factors were examined using Pearson’s χ^2^ tests and Mann–Whitney U-tests. Effect sizes were calculated for significant differences between CP-Ns and CP-Ps using Cohen’s d (Mann–Whitney U t-test; trivial: < 0.2; small: 0.2–0.5; moderate: 0.5–0.8; large. ≥ 0.8) or Cramer’s V (Pearson’s χ^2^ test; small: 0.06–0.15; moderate: 0.16–0.26; large: ≥ 0.26).

Logistic regression analyses were conducted to determine the effects of PA on susceptibility to COVID-19 infection; therefore, odds ratios (OR) and 95% confidence intervals (CI) were calculated. The covariates were age, sex, migration background, SES, chronic diseases, and smoking status. All analyses met the assumptions for the use of logistic regression analyses.

The level of statistical significance was set to *p* < 0.05. All analyses were performed using SPSS software version 29.0.

## Results

### Demographic Data

The sample had a mean age of 41.1 years (SD 14.2; range: 16 to 93 years) and included 64.3% females (Table 1). Overall, 214 subjects (4.1%) had a migration background, 4314 (81.3%) had a high SES, 956 (18.0%) had a middle SES and 38 (0.7%) had a low SES. Both migration background and SES were significantly different between CP-Ns and CP-Ps (migration background: *p* = 0.045; SES: *p* = 0.037). Furthermore, 4160 persons (78.7%) had no comorbidities, 547 (10.3%) had one comorbidity and 581 (11.0%) had two or more comorbidities. The CP-Ps were significantly more likely to have at least one chronic disease than the CP-Ns (*p* = 0.003). In addition, 1010 persons (21.4%) reported smoking prior to the pandemic. More CP-Ns were smokers than CP-Ps (*p* < 0.001).

**Table 1:** Sample characteristics

	Total (*n* = 5338)	CP-N (*n* = 4884)	CP-P (*n* = 454)	*p* value	Effect size
Mean age (years; mean and SD)	41.1 (14.2)	40.1 (14.2)	41.8 (14.9)	.684^a^	
Sex, n (%)				.891^b^	
Female	3401 (64.3%)	3111 (64.3%)	290 (64.6%)		
Male	1889 (35.7%)	1730 (35.7%)	159 (35.4%)		
Migration background, n (%)				**.045**^**b**^	.028^c^
Yes	214 (4.1%)	188 (3.9%)	26 (5.9%)		
No	5055 (95.9%)	4637 (96.1%)	418 (94.1%)		
SES, n (%)				**.037**^**b**^	.037^c^
High	4314 (81.3%)	3967 (81.7%)	347 (76.8%)		
Middle	956 (18.0%)	855 (17.6%)	101 (22.3%)		
Low	38 (0.7%)	34 (0.7%)	4 (0.9%)		
Comorbidity				**.003**^**b**^	.047^c^
No, n (%)	4160 (78.7%)	3825 (79.1%)	335 (74.4%)		
Yes, one, n (%)	547 (10.3%)	503 (10.4%)	44 (9.8%)		
Yes, two or more, n (%)	581 (11.0%)	510 (10.5%)	71 (15.8%)		
Smoking				**< .001**^**b**^	.056^c^
No, n (%)	3712 (78.6%)	3354 (77.9%)	358 (86.1%)		
Yes, n (%)	1010 (21.4%)	952 (22.1%)	58 (13.9%)		

p < .05 was defined as significant and marked in bold

*CP-N* contact person who stayed negative; *CP-P* contact person who tested positive; *SES* socioeconomic status

^a^Mann–Whitney U test

^b^Pearson’s χ^2^ test

^c^Cramer’s V

### Lifestyle

#### Physical Activity

**Fig. 2:**
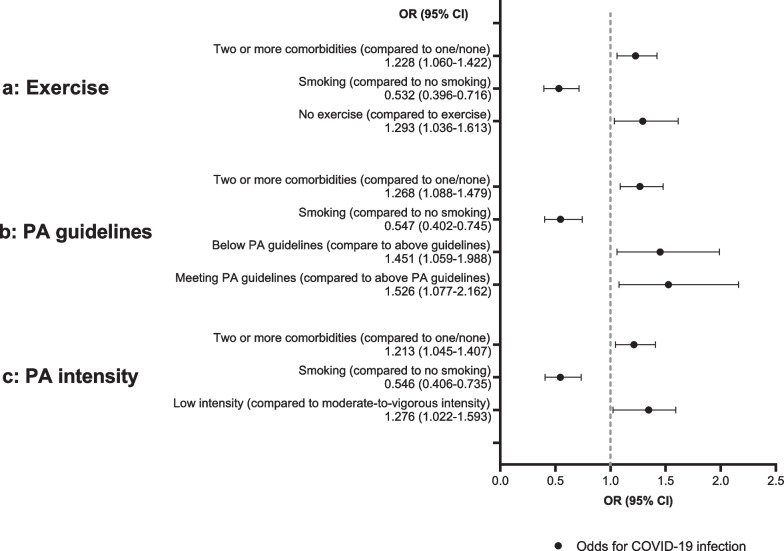
Risk factors for COVID-19 infection; binary logistic regression analyses, adjusted for age, sex, migration background, and socioeconomic status. **A** Exercise (no compared to yes); **B** PA guidelines (below and meeting PA guidelines compared to above PA guidelines); **C** PA intensity (low intensity vs. moderate-to-vigorous intensity). PA, physical activity

In total, 3658 persons (68.5%) reported being active before the pandemic (Table 2a). The average PA duration was 162.4 min/week (SD 194.1 min/week). The PA levels of most subjects were below guidelines (*n* = 2705, 56.7%). Significantly more CP-Ns were active before the pandemic than CP-Ps (69.1% vs. 62.6%; *p* = 0.004). Furthermore, CP-Ns reported more PA per week than CP-Ps (164.1 min/week vs. 143.2 min/week; *p* = 0.038), resulting in higher PA levels (above PA guidelines: 20.6% vs. 13.9%; meeting PA guidelines: 23.1% vs. 25.6%; below PA guidelines: 56.3% vs. 60.5%; *p* = 0.006). CP-Ns also performed higher intensity PA than CP-Ps (low intensity: 33.1% vs. 40.2%; moderate-to-vigorous intensity: 66.9% vs. 59.8%; *p* = 0.003). Controlling for demographics, chronic conditions, and smoking, the odds of becoming infected were significantly higher in inactive people (OR 1.293; 95% CI: 1.036–1.613; exercise (yes) as reference group; Table 3a, Fig. 2a). People who were below PA guidelines had 1.45-fold increased odds of infection compared with people above PA guidelines (95% CI: 1.059–1.998; Table 3b, Fig. 2b). People meeting PA guidelines had 1.53-fold increased odds of infection compared with people above PA guidelines (95% CI: 1.077–2.162, Table 3b, Fig. 2b). Also, people who reported low intensity had 1.28-fold increased odds of infection compared with those who reported moderate-to-vigorous intensity (95% CI: 1.022–1.593, Table 3c, Fig. 2c).

**Table 2:** Exercise, physical activity level, fitness, sedentary behaviour, and screen time before the pandemic

	Total (*n* = 5338)	CP-N (*n* = 4884)	CP-P (*n* = 454)	*p* value	Effect size
*(a) Exercise*					
Exercise before the pandemic, n (%)				**.004**^**a**^	**.039**^**b**^
Yes	3658 (68.5%)	3374 (69.1%)	284 (62.6%)		
No	1680 (31.5%)	1510 (30.9%)	170 (37.4%)		
PA duration, min/week, mean (SD)	162.4 (194.1)	164.1 (192.3)	143.2 (212.6)	**.005**^**c**^	**.041**^**d**^
METs, number/week, mean (SD)	6.7 (5.6)	6.8 (5.6)	6.1 (5.6)	**.004**^**c**^	**.040**^**d**^
PA level, n (%)				**.006**^**a**^	**.047**^**b**^
Above PA guidelines	956 (20.0%)	900 (20.6%)	56 (13.9%)		
Meeting PA guidelines	1113 (23.3%)	1010 (23.1%)	103 (25.6%)		
Below PA guidelines	2705 (56.7%)	2461 (56.3%)	244 (60.5%)		
PA intensity, n (%)				**.003**^**a**^	**.042**^**b**^
Moderate-to-vigorous intensity	3371 (66.3%)	3111 (66.9%)	260 (59.8%)		
Low intensity	1716 (33.7%)	1541 (33.1%)	175 (40.2%)		
*(b) Sedentary behaviour h/day, mean (SD)*					
Total sedentary behaviour	4.1 (3.5)	4.1 (3.5)	4.0 (3.4)	.797^c^	
Screen time	3.0 (2.8)	3.0 (2.8)	3.0 (2.9)	.774^c^	

p < .05 was defined as significant and marked in bold

*CP-N* contact person who stayed negative; *CP-P* contact person who tested positive; *PA* physical activity

^a^Pearson’s χ^2^ test

^b^Cramer’s V

^c^Mann–Whitney U test

^d^Cohen’s d

**Table 3:** Risk factors for COVID-19 infection

	B	Std. error	Sig.	OR	95% CI	
					Lower bound	Upper bound
*(a) Exercise*						
Nagelkerke Pseudo R-Square 1.9%						
Age (years)	− .001	.004	.893	.999	.992	1.007
Sex (male vs. female)	− .036	.112	.744	.964	.774	1.200
Migration background (yes)	.334	.232	.149	1.397	.887	2.200
SES (high vs. middle/low)	− .225	.121	.063	.799	.630	1.012
Comorbidity (two or more vs. one/none)	.205	.075	**.006**	1.228	1.060	1.422
Smoking (yes)	.631	.151	**< .001**	.532	.396	.716
Exercise (no)	.257	.113	**.023**	1.293	1.036	1.613
Exercise (yes)	Ref.					
*(b) PA level*						
Nagelkerke Pseudo R-Square 2.0%						
Age (years)	− .001	.004	.743	.999	.991	1.007
Sex (male vs. female)	.011	.118	.928	1.011	.803	1.272
Migration background (yes)	.309	.247	.211	1.362	.839	2.210
SES (high vs. middle/low)	− .205	.127	.105	.814	.635	1.044
Comorbidity (two or more vs. one/none)	.238	.078	**.002**	1.268	1.088	1.479
Smoking (yes)	− .603	.158	**< .001**	.547	.402	.745
Below PA guidelines	.372	.161	**.021**	1.451	1.059	1.988
Meeting PA guidelines	.422	.178	**.018**	1.526	1.077	2.162
Above PA guidelines	Ref.					
*(c) PA intensity*						
Nagelkerke Pseudo R-Square 1.8%						
Age (years)	− .001	.004	.819	.999	.992	1.007
Sex (male vs. female)	− .030	.113	.791	.970	.778	1.211
Migration background (yes)	.369	.233	.113	1.446	.917	2.281
SES (high vs. middle/low)	− .218	.123	.075	.804	.632	1.022
Comorbidity (two or more vs. one/none)	.193	.076	**.011**	1.213	1.045	1.407
Smoking (yes)	− .605	.151	**< .001**	.546	.406	.735
Low intensity	.244	.113	**.031**	1.276	1.022	1.593
Moderate-to-vigorous intensity	Ref.					

p < .05 was defined as significant and marked in bold

Binary logistic regression analyses

A: exercise, yes as reference; B: above PA guidelines as reference; C: moderate-to-vigorous intensity as reference

#### Sedentary Behaviour

The average total sedentary behaviour was 4.1 h/day (SD 3.5 h/day), of which screen time was 3.0 h/day (SD 2.8 h/day; Table 2c). Neither average total sedentary behaviour nor screen time was significantly different between the two groups.

## Discussion

To our knowledge, this is the first study analysing the association between exercise and COVID-19 infection in unvaccinated persons who had a confirmed relevant contact with an infected individual. We found that CPs who reported being consistently physically active prior to the pandemic had 29% lower odds of being infected with COVID-19 than inactive CPs. In addition, our data indicated reduced odds of infection with increased PA levels. Specifically, the odds of COVID-19 infection decreased by 53% and 45%, respectively, in CPs whose PA levels were below 150 min and between 150 and 300 min, respectively, compared with CPs whose PA levels were greater than 300 min/week. Furthermore, the odds of infection increased by 28% among CPs who reported performing low intensity pre-pandemic compared with those who reported moderate-to-vigorous intensity.

These findings are consistent with those of other studies examining PA and risk of infection. For example, PA lowered the risk of COVID-19 infection by 11% in a systematic review and meta-analysis [12]. Furthermore, Cunningham found, at county level, that PA was negatively associated with the number of COVID-19 cases per 100,000 inhabitants [27].

The underlying mechanisms behind these findings can only be speculated. Data on the risk of upper respiratory tract infection suggest that PA increases the circulation of immunoglobulins, neutrophils, and natural killer cells, thereby enhancing immune response [28]. Although levels decrease a few hours after the end of a PA session, PA induces overall increased immunosurveillance of pathogens and thereby lowers susceptibility to infection. Along these lines, a systematic review and meta-analysis showed that regular moderate-to-vigorous PA resulted in a 31% lower prospective risk of upper respiratory tract infection [29].

In addition to improved immunosurveillance, PA has beneficial effects on systemic inflammation and thus on preventing the development of chronic diseases [30]; in our model, it influenced the odds of infection with COVID-19 alongside lifestyle factors. To date, most studies have focused on the association between comorbidities and patients’ COVID-19 outcomes. For example, a systematic review and meta-analysis included 16 studies investigating various comorbidities as potential risk factors for an infection [31]. The results indicated only obesity as a risk factor; schizophrenia, dementia, and coronary heart disease resulted in lower odds of infection. Other comorbidities, such as hypertension, high cholesterol, and diabetes, had no significant influence on infection. Nevertheless, data in the field remain ambiguous, and further studies are needed to clarify the association between individual diseases and the risk of infection.

Surprisingly, our data showed that individuals who smoked before the pandemic had a lower risk of infection than non-smokers. Similarly, another review and meta-analysis showed that, although smokers were more likely to present for testing, they were less likely to test positive for SARS-CoV-2 [32]. Its authors discussed the methods that led to this result critically. Among other factors, an inaccurate recording of smoking status was possible, whereby ex-smokers were potentially counted as non-smokers. A change in the sensitivity of COVID-19 testing methods and/or impaired access to or lower prioritisation within the healthcare system were other possible contributing factors. Still, the extent to which these factors may explain the findings is unclear.

### Strengths and Limitations

The major strength of this study is the large sample size, which was acquired using systematic data collection by the largest public health department in Germany. As no authorised vaccine was available at the time of the survey, the data are free of any bias that may have resulted from vaccination. Moreover, the sample consisted entirely of clearly defined CPs who had confirmed exposure to the virus.

Regardless, the study has several limitations. First, the data are based on self-reporting which could have led to answering based on social desirability, misreporting or overestimating PA levels. With about 43% of the sample achieving the WHO recommendations on PA, the data resemble that of the German Health Update by the Robert Koch Institute. According to this data, about 45% of females and 51% of males achieved the WHO PA recommendations in force at the time of at least 150 min/week [33]. Secondly, the data are limited by being confined to a regional cohort, as well as the questionnaires being mainly completed by individuals with high SES. We are aware that the assessment of SES is at risk of being biased, as people with lower SES might have poorer access to the internet or fewer skills with which to answer the survey. Thirdly, answering the questionnaire took about 30 min. Therefore, answering fatigue cannot be ruled out. Nevertheless, the amount of completely answered questionnaires is almost 60%. Fourthly, due to the adjustments of lockdowns and reopening of sports facilities during the course of the pandemic, we referred to the pre-pandemic data. This temporal distance might have led to misreporting or changes in behaviour as a result of the pandemic [34]. It remains unclear whether current lifestyle has a more sensitive effect on the risk of infection than pre-pandemic status.

Furthermore, adherence to quarantine measures, which was not investigated here, might have had an important impact on infection risk. However, analyses of this dataset concerning adherence have been published previously [35]. Finally, it must be emphasized that at this stage of the pandemic, nearly 70% of the world population has received at least one dose of a COVID-19 vaccine [36], and that vaccination was one of the most important pandemic control measures.

Nevertheless, our data show that PA had an impact on the odds of infection independently of vaccination. Still, the models were explained only to a small extent by PA or fitness; further studies are required to examine the risk factors.

## Conclusion

Having considered the limitations, we conclude that the data indicate that pre-pandemic, self-reported PA (especially for at least 300 min/week and at moderate-to-vigorous intensity) was associated with lower odds of COVID-19 infection in unvaccinated CPs. Other than smoking status and comorbidities, PA was the strongest modifiable risk factor for infection. Although the long-term effects of PA can only be speculated, inactive persons and persons with chronic conditions should be encouraged in the context of medical examinations and check-ups to adopt a more active lifestyle.

## Supplementary Information

**Additional file 1**. Online survey of the first wave of the CoCo-Fakt cohort study.

## Data Availability

The datasets generated during and/or analysed during the current study are available from the corresponding author on reasonable request.

## Abbreviations

CoCo-Fakt: Cologne-Corona-Beratung und Unterstützung Für Index- und KontAKt-Personen während der Quarantäne-ZeiT (Cologne-Corona counselling and support for index and contacts during the quarantine period—author’s translation)
COVID-19: Coronavirus disease
CP: Contact person
CP-P: Contact person who tested positive
CP-N: Contact person who stayed negative
DiKoMa: Digitales Kontaktmanagement (digital contact management)
DEGS: German Health Interview and Examination Survey for Adults
IP: Infected person
MET: Metabolic equivalent
NCD: Non-communicable disease
PA: Physical activity
SARS-CoV-2: Severe acute respiratory syndrome coronavirus type 2
SES: Socioeconomic status
