# Supplementary material for: Self-Reported Pre-Pandemic Physical Activity and Likelihood of COVID-19 Infection: Data from the First Wave of the CoCo-Fakt Survey
Source: Sports Med Open. 2023 Jun 21;9:48. doi: 10.1186/s40798-023-00592-6 (PMC10284734; doi:10.1186/s40798-023-00592-6)
Supplement: Supplementary file 1 — Additional file 1. Online survey of the first wave of the CoCo-Fakt cohort study. [file 40798_2023_592_MOESM1_ESM.pdf]

Additional file 1. Online survey of the first wave of the CoCo-Fakt cohort study

Online survey

Information on the study (sub-projects A, B, G)

CoCo-Fakt – Cologne-Corona-Beratung und Unterstützung Für Index- und KontAKt-Personen während der Quarantäne-ZeiT (Cologne-Corona counselling and support for index patients and contacts during the quarantine period)

Thank you for your interest in the survey. Following the provided link, you have arrived at our survey on the online platform. Your participation in the study is voluntary. All information that you provide is anonymous. Only your email address was used to process this online procedure. It will be irretrievably deleted after your participation. However, before you decide for or against participating, you should understand why this survey is being conducted and what is involved. Please take the time to read the following information carefully and decide whether or not you want to participate. If you have any questions about the survey, you can always contact the persons mentioned in the link to the privacy policy. They will answer your questions as soon as possible.

Dear study participant,

Since the first infections with the COVID-19 virus were described in Wuhan in December 2019, it has spread worldwide within a very short time and led to considerable restrictions in the lives of all people. To date, little is known about the care and course of the disease during the domestic isolation or quarantine period. This includes not only the patients themselves, but also contact persons of the first order who have been designated as contact persons according to the Infection Protection Act (IfSG). Due to the duration of possible restrictive measures, it is therefore relevant to investigate the respective approaches and creative solutions in relation to their personal situation, and to elaborate which factors are important/have an impact on the course of the disease.
The purpose of this online survey is therefore to gain knowledge about relevant aspects of the COVID-19 infection and/or the quarantine/domestic isolation. Understanding the handling of the consequences on a medical as well as psychosocial level is necessary – even if some time has passed since the quarantine, to generate recommendations for the proceeding months.
The questionnaire includes questions regarding your current situation, how you deal with it, your lifestyle and personal assessment as well as that of your children or – if applicable, of your pregnancy. It was developed on the basis of the World Health Organization's COVID-19 Snapshot Monitoring (COSMO) survey. Answering the questions will take about 30 minutes.

Privacy Policy

The city's health department collects personal data from you for the purpose of scientific monitoring. The protection of your personal data is a central concern. Accordingly, we feel obliged to comply with the legal requirements, in particular the European Basic Data Protection Regulation (hereinafter referred to as “DSGVO”), the Data Protection Act of the State of North Rhine-Westphalia (DSG NRW), the Infection Protection Act (IfSG) and the Higher Education Act of the State of North Rhine-Westphalia.

Please follow this link to participate in the survey in Turkish:

Anketin türkce versiyonuna katılmak istiyorum.

Please follow this link to participate in the survey in German:

Ich möchte an der deutschen Version des Fragebogens teilnehmen.

1) Date of completion: _______________(day/month/year)

Personal Background

Please answer the following personal information

2) What is your age? _______________ years

3) What is your sex?

- o Female
- o Male
- o Other

4) Which language do you mainly speak? _______________

5) How many school years did you complete? _______________

6) What is your highest professional qualification?
Please include degrees obtained in foreign countries by selecting an equivalent German degree.

- o Job training, professional internship
- o Job preparation year
- o Apprenticeship, dual system vocational training
- o Vocational qualification at a technical college, preparatory college for intermediate service in public administration
- o Training centres/schools for health and social professions: One-year Programmes (e.g. nursing assistant, geriatric nurse, paramedic)
- o Training centres/schools for health and social professions: Two-year Programmes (e.g. massage therapist, hydro therapist, Pharmaceutical Technical Assistant (PTA), podiatrist)
- o Training centres/schools for health and social professions: Three-year Programmes (e.g. physiotherapy, health and nursing care, Medical Technician (MTA), geriatric care)
- o Training centres/schools for educators
- o Master craftsman, technician or equivalent technical college degree
- o Technical school of the DDR
- o Specialist academy (“Fachakademie”, only in Bavaria)
- o University/technical college degree
- o Other: _______________

7) **Do you have any chronic diseases?**

- Yes
- No
- I don’t know

8) **If yes, do you have any of the following diseases?**
You can select multiple answers.

- Asthma, including allergic asthma
- Chronic bronchitis, chronic obstructive pulmonary disease, pulmonary emphysema
- Heart attack, coronary heart disease
- Congestive heart failure
- Stroke
- Diseases of the musculoskeletal system
- Diabetes -- type 1 or type 2 (excluding gestational diabetes)
- Hypertension/high blood pressure
- Hypercholesterolemia
- Allergies, such as hay fever, allergic reactions of the eyes or skin, food allergies or other allergies (excluding allergic asthma)
- Chronic liver diseases
- Chronic kidney problems or kidney failure
- Depression
- Cancer, which type? _______________
- Inflammatory bowel disease (Crohn's disease, ulcerative colitis)
- Other: _______________

9) **Are you in a relationship (including marriage)?**

- Yes
- No

10) **Do you have children?**

- Yes
- No

11) **If yes, do you have children under the age of 3?**

- Yes
- No (Please continue with question 22)

You have stated that you have a child/children under the age of 3. Please think of your child/children in this age group now.

12) How many children under the age of 3 do you have?

Number of boys: _______________
Number of girls: _______________
Number of others: _______________

13) Does your child / do your children under the age of 3 have any chronic diseases?
If you have more than one child of this age group, please enter the respective diseases for the respective sex and indicate the number of sick children.

- o No
- o **Boys**: Yes, the following: _______________
- o **Girls**: Yes, the following: _______________
- o **Other**: Yes, the following: _______________

14) How many of your children under the age of 3 were in quarantine? _______________

15) How did your child/children feel during the quarantine period?

	**Not at all or less than 1 day**					**Always or every day**
The child/children felt nervous, anxious, or on edge.	O	O	O	O	O	O
The child/children felt depressed.	O	O	O	O	O	O
The child/children felt lonely.	O	O	O	O	O	O
Thoughts about its/their experiences during the Coronavirus pandemic cause it/them to have physical reactions, such as sweating, trouble breathing, nausea or a pounding heart.	O	O	O	O	O	O

16) Did your child/children visit a kindergarten or day care centre before the contact ban on March 16?

- o Yes
- o No

17) How did you handle the lack of childcare (e.g. kindergarten/day care)?

____
____

18) How often did your child/children meet social contacts outside the immediate family (grandparents, friends) before the contact ban on March 16?

- Not at all
- Once every 2 weeks
- Once per week
- Several times per week
- Daily

19) How did you handle the lack of social contacts (e.g. grandparents, friends) of your child/children?

____
____

20) Did your child/children regularly visit a (sports) club or music school before the contact ban on March 16?

- No
- Yes, a (sports) club
- Yes, a music school
- Yes, private music lessons
- Yes, other: ___________________________

21) How did you handle the lack of leisure activities?

____
____

These questions were extended analogously to the following age groups:

3 to under 6 years
6 to under 10 years
10 to under 14 years
14 to under 16 years

From the age group of 6 years old children (and older) the terms kindergarten and day care were replaced by school

Housing situation

Please enter the following information about your housing situation.

22) Does your house/apartment have...

- ○ a garden
- ○ a balcony or terrace
- ○ both
- ○ neither

23) How many rooms does your home have? _______________
Please note: Kitchen, hallway and bathroom do not count as rooms.

24) Do you live alone?
Please note: This refers to the household where you spent most of your quarantine.

- ○ Yes (Please continue with question 28)
- ○ No

25) How many adults permanently live in your household (other than you)? _______________

26) Is it possible in your household to be alone in a room during quarantine?

- ○ Yes
- ○ No

27) Did you have to isolate yourself from your partner/your children/your family during quarantine?

- ○ Yes, in a shared apartment
- ○ Yes, in a separate apartment
- ○ No, because: _______________

Your quarantine situation

28) Why did you have to quarantine?

- ○ I tested positive for the coronavirus.
- ○ I was a contact person. (Please continue with question 30)
- ○ I was a contact person several times. (Please continue with question 30)
- ○ I was a contact person and was tested positive for the coronavirus afterwards. (Please continue with question 30)
- ○ I was a travel returnee. (Please continue with question 43)
- ○ Other: _______________ (Please continue with question 43)
- ○ I don't know. (Please continue with question 43)

29) How would you personally describe the course of the disease after quarantine?

- Completely symptom-free
- Mild symptoms
- 1-3 days of noticeable symptoms
- Severe feeling of sickness
- Disease-related anxiety
- Other: _______________

30) Do you have a suspicion how you might have been infected?

- Yes
- No (Please continue with question 40)

If yes, we would like to ask you about the situation in which you may have been infected / had contact with a coronavirus patient.

31) I may have been infected / had contact with a coronavirus patient in the following situation:
Please indicate the situation that you think was most likely.

- With family members who live with me
- With family members who do not live with me
- With friends or acquaintances (not at a celebration / party)
- At work
- At school / university / day care
- I received medical treatment (doctor's office, hospital, rehabilitation clinic)
- On public transport (bus / train / tram)
- While shopping
- During sport (in a club / in the gym)
- At a celebration / party / family celebration with approx. ________ people
- At a concert
- In a restaurant / bar / café
- When singing (e.g. in a choir)
- In a religious institution (church / mosque)
- Other: _______________

32) Did the person you had contact with wear a mask?

- Community mask
- Medical face mask
- FFP2 mask
- FFP3 mask
- Other: _______________
- No
- I don't know

33) I wore the following protection during the contact:
You can select multiple answers.

- ○ None
- ○ Community mask
- ○ Medical face mask
- ○ FFP2 mask
- ○ FFP3 mask
- ○ Protective gown
- ○ Gloves
- ○ Other: _______________
- ○ I don't know

34) The contact took place within enclosed spaces:

- ○ Yes
- ○ No

35) The total time of contact was (added up): _______________ min

36) The distance during contact was: _______________ m

37) If the contact took place at the workplace: What applies to you?

- ○ I work as a healthcare professional (medical staff)
- ○ I work as a healthcare professional (nursing staff; nursing home / outpatient nursing service)
- ○ I work in an office with _______________ other people
- ○ I work in the retail sector
- ○ I work in a school / day care centre
- ○ I work in the following field: _______________

38) If the contact took place on public transport (bus / train / tram): How often do you use public transport per week?

- ○ 1-2 days per week
- ○ 3-4 days per week
- ○ 5-7 days per week

39) If the contact took place within enclosed spaces: How regularly was the room aired out?

- ○ Permanently
- ○ Several times per hour
- ○ Every 1 to 2 hours
- ○ Less than every 2 hours
- ○ There was a ventilation system
- ○ Not at all
- ○ I do not know (anymore)

Your quarantine situation (continued)

40) Have you quarantined more than once?
- o Yes
- o No

41) If you quarantined only once: How long did you quarantine for?
From: _______________ (day/month/year) To: _______________ (day/month/year)

42) If you quarantined more than once: When was the first time you quarantined?

From: _______________ (day/month/year) To: _______________ (day/month/year)

When did you quarantine again?

From: _______________ (day/month/year) To: _______________ (day/month/year)

When did you quarantine again?

From: _______________ (day/month/year) To: _______________ (day/month/year)

Knowledge of the quarantine recommendations – Part I

In this section we would like to find out how you got information about the recommendations for dealing with the quarantine and to what extent you were able to implement them.

43) Please indicate to what extent you agree with the following statements.

	I fully agree					I do not agree at all
1. I think the new coronavirus is dangerous.	O	O	O	O	O	O
2. It was clearly explained to me why I should go into quarantine.	O	O	O	O	O	O
3. It was clearly explained to me how I should behave in quarantine.	O	O	O	O	O	O
4a. I think the quarantine measures are too strict.	O	O	O	O	O	O
4b. I think the quarantine measures are too loose.	O	O	O	O	O	O
5a. When I quarantine, I protect myself.	O	O	O	O	O	O
5b. When I quarantine, I protect other members of my household.	O	O	O	O	O	O
5c. If I quarantine, I will protect our society from the further spread of the coronavirus.	O	O	O	O	O	O

6. People in my professional and social environment expected me to implement the quarantine measures.	O	O	O	O	O	O
7. During my quarantine, I had difficulties providing myself with the things I needed for everyday life (e.g. groceries, drugstore items).	O	O	O	O	O	O

Knowledge of the quarantine recommendations – Part II

1) ***Recommendation: Do not leave your apartment/house.***

Were you aware of this recommendation during your quarantine? Yes □ No □

	**I fully implemented it**					**I did not implement it at all**
Did you implement this recommendation?	O	O	O	O	O	O
	**I did not find it difficult at all**					**It was very difficult for me**
How difficult did you find it to implement this recommendation?	O	O	O	O	O	O

2) ***Recommendation: Do not allow visitors into your home.***

Were you aware of this recommendation during your quarantine? Yes □ No □

	**I fully implemented it**					**I did not implement it at all**
Did you implement this recommendation?	O	O	O	O	O	O
	**I did not find it difficult at all**					**It was very difficult for me**
How difficult did you find it to implement this recommendation?	O	O	O	O	O	O

3) ***Recommendation: Avoid personal contact with postmen and delivery services. If contact is necessary, wear a face mask.***

Were you aware of this recommendation during your quarantine? Yes □ No □

	**I fully implemented it**					**I did not implement it at all**
Did you implement this recommendation?	O	O	O	O	O	O
	**I did not find it difficult at all**					**It was very difficult for me**
How difficult did you find it to implement this recommendation?	O	O	O	O	O	O

The following questions are only addressed to those participants who do not live alone. If you live alone, please continue with question 44.

4) ***Recommendation Stay separate from other household members in a separate room.***

Were you aware of this recommendation during your quarantine? Yes □ No □

	**I fully implemented it**					**I did not implement it at all**
Did you implement this recommendation?	O	O	O	O	O	O
	**I did not find it difficult at all**					**It was very difficult for me**
How difficult did you find it to implement this recommendation?	O	O	O	O	O	O

5) ***Recommendation: Sleep separately from other household members in a separate room. If this is not possible, sleep in a separate bed with at least one metre distance to the next bed.***

Were you aware of this recommendation during your quarantine? Yes □ No □

	**I fully implemented it**					**I did not implement it at all**
Did you implement this recommendation?	O	O	O	O	O	O
	**I did not find it difficult at all**					**It was very difficult for me**
How difficult did you find it to implement this recommendation?	O	O	O	O	O	O

6) ***Recommendation: Only have contact with other household members if you need their help.***

Were you aware of this recommendation during your quarantine? Yes □ No □

	**I fully implemented it**					**I did not implement it at all**
Did you implement this recommendation?	O	O	O	O	O	O
	**I did not find it difficult at all**					**It was very difficult for me**
How difficult did you find it to implement this recommendation?	O	O	O	O	O	O

7) ***Recommendation: Keep a distance of at least 1.5m to other household members.***

Were you aware of this recommendation during your quarantine? Yes □ No □

	**I fully implemented it**					**I did not implement it at all**
Did you implement this recommendation?	O	O	O	O	O	O
	**I did not find it difficult at all**					**It was very difficult for me**
How difficult did you find it to implement this recommendation?	O	O	O	O	O	O

8) ***Recommendation: Wear a face mask when in contact with other household members.***

Were you aware of this recommendation during your quarantine? Yes □ No □

	**I fully implemented it**					**I did not implement it at all**
Did you implement this recommendation?	O	O	O	O	O	O
	**I did not find it difficult at all**					**It was very difficult for me**
How difficult did you find it to implement this recommendation?	O	O	O	O	O	O

9) ***Recommendation: Take your meals in a different room than the other household members.***

Were you aware of this recommendation during your quarantine? Yes □ No □

	**I fully implemented it**					**I did not implement it at all**
Did you implement this recommendation?	O	O	O	O	O	O
	**I did not find it difficult at all**					**It was very difficult for me**
How difficult did you find it to implement this recommendation?	O	O	O	O	O	O

10) ***Recommendation: Use the bathroom, hallway, kitchen and other common rooms only when absolutely necessary.***

Were you aware of this recommendation during your quarantine? Yes □ No □

	**I fully implemented it**					**I did not implement it at all**
Did you implement this recommendation?	O	O	O	O	O	O
	**I did not find it difficult at all**					**It was very difficult for me**
How difficult did you find it to implement this recommendation?	O	O	O	O	O	O

11) **Does your household have more than one toilet? Yes □ No □**

If yes, Recommendation: Use only one of the toilets. Other members of the household should not use this toilet.

Were you aware of this recommendation during your quarantine? Yes □ No □

	**I fully implemented it**					**I did not implement it at all**
Did you implement this recommendation?	O	O	O	O	O	O
	**I did not find it difficult at all**					**It was very difficult for me**
How difficult did you find it to implement this recommendation?	O	O	O	O	O	O

12) ***Recommendation: The bathroom you use should be cleaned at least once a day.***

Were you aware of this recommendation during your quarantine? Yes □ No □

	**I fully implemented it**					**I did not implement it at all**
Did you implement this recommendation?	O	O	O	O	O	O
	**I did not find it difficult at all**					**It was very difficult for me**
How difficult did you find it to implement this recommendation?	O	O	O	O	O	O

13) ***Recommendation: Surfaces frequently touched by you (bedside table, door handles, smartphone, work surfaces ...) should be cleaned once a day.***

Were you aware of this recommendation during your quarantine? Yes □ No □

	**I fully implemented it**					**I did not implement it at all**
Did you implement this recommendation?	O	O	O	O	O	O
	**I did not find it difficult at all**					**It was very difficult for me**
How difficult did you find it to implement this recommendation?	O	O	O	O	O	O

14) ***Recommendation: Air all rooms regularly.***

Were you aware of this recommendation during your quarantine? Yes □ No □

	**I fully implemented it**					**I did not implement it at all**
Did you implement this recommendation?	O	O	O	O	O	O
	**I did not find it difficult at all**					**It was very difficult for me**
How difficult did you find it to implement this recommendation?	O	O	O	O	O	O

15) ***Recommendation: Sneeze into the crook of your arm or use a disposable tissue.***

Were you aware of this recommendation during your quarantine? Yes □ No □

	**I fully implemented it**					**I did not implement it at all**
Did you implement this recommendation?	O	O	O	O	O	O
	**I did not find it difficult at all**					**It was very difficult for me**
How difficult did you find it to implement this recommendation?	O	O	O	O	O	O

16) ***Recommendation: Wash your hands regularly for at least 20 seconds, particularly after blowing your nose or sneezing.***

Were you aware of this recommendation during your quarantine? Yes □ No □

	**I fully implemented it**					**I did not implement it at all**
Did you implement this recommendation?	O	O	O	O	O	O
	**I did not find it difficult at all**					**It was very difficult for me**
How difficult did you find it to implement this recommendation?	O	O	O	O	O	O

17) ***Recommendation: Keep used tissues, gloves and other waste in a lidded bin in your room.***

Were you aware of this recommendation during your quarantine? Yes □ No □

	**I fully implemented it**					**I did not implement it at all**
Did you implement this recommendation?	O	O	O	O	O	O
	**I did not find it difficult at all**					**It was very difficult for me**
How difficult did you find it to implement this recommendation?	O	O	O	O	O	O

18) ***Recommendation: After washing your hands, use paper towels or a towel that is only used by you and change it daily.***

Were you aware of this recommendation during your quarantine? Yes □ No □

	**I fully implemented it**					**I did not implement it at all**
Did you implement this recommendation?	O	O	O	O	O	O
	**I did not find it difficult at all**					**It was very difficult for me**
How difficult did you find it to implement this recommendation?	O	O	O	O	O	O

19) ***Recommendation: Wash your clothes at a minimum of 60 degrees and separately from the laundry of other household members.***

Were you aware of this recommendation during your quarantine? Yes □ No □

	**I fully implemented it**					**I did not implement it at all**
Did you implement this recommendation?	O	O	O	O	O	O
	**I did not find it difficult at all**					**It was very difficult for me**
How difficult did you find it to implement this recommendation?	O	O	O	O	O	O

44) **If you generally found it difficult to implement the quarantine measures: Exactly what made the implementation difficult for you?**
Please name the aspects that you consider most important.

1) I found the implementation difficult because________________________________
2) I found the implementation difficult because________________________________
3) I found the implementation difficult because________________________________

Mental Situation

In this section we would like to know how you felt during the quarantine period and how you and others could be supported during this phase.

Please think back to the last four weeks before the contact ban was implemented on March 16.

45) **Please answer the following questions according to your situation before the contact ban is implemented on March 16.**

	Yes	No
a) During the past month, have you often been bothered by feeling down, depressed, or hopeless?	O	O
b) During the past month, have you often been bothered by little interest or pleasure in doing things?	O	O

46) **Please indicate how much you agree with the following statements regardless of the quarantine situation.**

	I fully agree					I do not agree at all
I tend to bounce back quickly after hard times.	O	O	O	O	O	O
I have a hard time making it through stressful events.	O	O	O	O	O	O
It does not take me long to recover from a stressful event.	O	O	O	O	O	O
It is hard for me to snap back when something bad happens.	O	O	O	O	O	O
I usually come through difficult times with little trouble.	O	O	O	O	O	O
I tend to take a long time to get over set-backs in my life.	O	O	O	O	O	O
I am generally satisfied with my life situation.	O	O	O	O	O	O

47) **Below you will find descriptions of how you may have mostly felt or behaved during the quarantine period.**
Please select the answer that best matches/matched your state of health during this phase.

	Not at all or less than 1 day					Always or every day
I felt nervous, anxious, or on edge.	O	O	O	O	O	O
I felt depressed.	O	O	O	O	O	O
I felt lonely.	O	O	O	O	O	O
I felt hopeful about the future.	O	O	O	O	O	O
Thoughts about my experiences during the	O	O	O	O	O	O

Coronavirus pandemic caused me to have physical reactions, such as sweating, trouble breathing, nausea or a pounding heart.						

48) How much do/did the following statements apply to you during the quarantine period?

	**Fully applicable**					**Not applicable at all**
I receive support offers from family, friends or neighbours.	O	O	O	O	O	O
I have a plan for my daily routine in terms of sleep, work, or physical activities.	O	O	O	O	O	O
I discover activities that make it easier for me to stay at home.	O	O	O	O	O	O
I make phone calls or exchange information with family, friends and acquaintances via digital media.	O	O	O	O	O	O
I am bored.	O	O	O	O	O	O
I cannot do anything to influence the situation in a positive way.						

49) Please answer the following questions regarding your experience during the quarantine period.

	**I fully agree**					**I do not agree at all**
I always find ways for myself to continue.	O	O	O	O	O	O
I know that I will not allow myself to be discouraged.	O	O	O	O	O	O
I learn important and useful lessons for my life.	O	O	O	O	O	O
I learn ways to better cope with it next time.	O	O	O	O	O	O

50) What helped you most during the quarantine period?
Please name the aspects that you consider most important.

1) What helped me the most was ________________________________
2) What helped me the most was ________________________________
3) What helped me the most was ________________________________

51) What did you worry about most during the quarantine period?
Please name the aspects that you consider most important.

1) What I worried about most was ________________________________
2) What I worried about most was ________________________________
3) What I worried about most was ________________________________

52) Did your quarantine result in financial losses or did you have higher financial expenses than usual?
Please name the aspects that you consider most important.

1) I had financial losses/higher financial expenses because _____________________
   ____
2) I had financial losses/higher financial expenses because _____________________
   ____
3) I had financial losses/higher financial expenses because _____________________
   ____

53) Did you make use of professional support systems, such as helplines?

- o Yes, the following: _____________________
- o No (Please continue with question 55)

54) If so, would you recommend them to others?

- o Yes, because _____________________
- o No, because _____________________

55) Which further measures would you like to see or would you have liked to see?
Please name the aspects that you consider most important.

1) I would like/would have liked________________________________
2) I would like/would have liked________________________________
3) I would like/would have liked________________________________

56) Were you excluded due to your quarantine period?
a) In the professional environment

- o Yes
- o Partly
- o No

b) In your private environment

- o Yes
- o Partly
- o No

Eating behaviour during the quarantine period

57) Which meal would you consider your main meal?

- o Breakfast
- o Lunch
- o Dinner
- o Other: ____________________

58) Did anything change with regard to your meals during the quarantine period?

- o Yes
- o No (Please continue with question 60)

59) If yes, what changed with regard to your meals during the quarantine period?

	Yes	No	Partly
Eating healthier	O	O	O
Eating less healthy	O	O	O
More snacks between meals	O	O	O
More nibbling (e.g. while watching TV, working etc.)	O	O	O
Other: _______________________________	O	O	O

60) How often do you consume the following foods each week?

	Several times per day	Once per day	Several times per week	Rarely	Never
Sweet food (e.g. chocolate, gummi bears etc.)	O	O	O	O	O
Salty food (e.g. chips, nuts etc.)	O	O	O	O	O
Fresh vegetables	O	O	O	O	O
Fresh fruit	O	O	O	O	O

61) Did anything change regarding the food you eat during the quarantine period?

- Yes
- No (Please continue with question 63)

***62)* If yes, what changed during the quarantine period with regard to the food you eat?**
You can select multiple answers.

- I eat more
- I eat less.
- I eat more sweets.
- I eat less sweets.
- I eat more salty snacks.
- I eat less salty snacks.
- I eat more fruit.
- I eat less fruit.
- I eat more vegetables.
- I eat less vegetables.
- I eat more consciously.
- I eat less consciously.
- I eat healthier.
- I eat less healthy.
- Other: ____________________

63) Did your body weight change during the quarantine period?

- Yes
- No (Please continue with question 65)

64) If yes, how did your body weight change during the quarantine period?

- I lost ____________ kg.
- I gained ____________ kg.

Exercise and relaxation

In this section we would like to know if and how it was possible to adapt your usual lifestyle to the quarantine period. Therefore, we will first ask you about your usual activities before the Corona pandemic in order to compare them with the changes.

65) Were you active in sports before the Corona period? Yes □ No □

Type of exercise	How often per week?	For how many minutes each?	Indepen-dently	In a club	In a gym and/or with a coach	Media-/online-based offer	Other
1. ____________________ Intensity: □ very light □ light □ moderate □ vigorous □ very vigorous	_____	_____	O	O	O	O	O
2. ____________________ Intensity: □ very light □ light □ moderate □ vigorous □ very vigorous	_____	_____	O	O	O	O	O
3. ____________________ Intensity: □ very light □ light □ moderate □ vigorous □ very vigorous	_____	_____	O	O	O	O	O

66) Did you regularly do relaxation activities (e.g. yoga, meditation) before the corona pandemic? Yes □ No □

Relaxation activity	How often per week?	For how many minutes each?	Indepen-dently	In a club	In a gym and/or with a coach	Media-/online-based offer	Other
1. ____________________	_____	_____	O	O	O	O	O
2. ____________________	_____	_____	O	O	O	O	O
3. ____________________	_____	_____	O	O	O	O	O

67) On average, how many hours per week did you usually spend sitting <u>before</u> the corona pandemic? (e.g. in the car, on the sofa, while watching TV, ...)

**At work**		**In your private environment**	
Office work without screen work	☐ h	Computer/phone/tablet:	☐ h
Office work with screen work (e.g. computer):	☐ h	TV	☐ h
Driving a car:	☐ h	Driving a car:	☐ h
Other: ____________________	☐ h	Reading/writing	☐ h
Other: ____________________	☐ h	Hobbies (sewing, handicrafts, etc.):	☐ h
	.	Other: ____________________	☐ h

Lifestyle during the quarantine period

68) How did you feel <u>during</u> the quarantine period?

	**Fully applicable**					**Not applicable at all**
I feel/felt limited in my physical performance.	O	O	O	O	O	O
I feel/felt exhausted.	O	O	O	O	O	O
I feel/felt tired.	O	O	O	O	O	O
I always feel/felt physically fit.	O	O	O	O	O	O

69) Were you active in sports <u>during</u> the quarantine period? Yes □ No □

**Type of exercise**	**How often per week?**	**For how many minutes each?**	**Indepen-dently**	**In a club**	**In a gym and/or with a coach**	**Media-/online-based offer**	**Other**
1. ____________________<br><u>Intensity:</u> □ very light □ light □ moderate □ vigorous □ very vigorous	_____	_____	O	O	O	O	O

2. ____________________<br>Intensity: □ very light □ light □ moderate □ vigorous □ very vigorous	_____	_____	O	O	O	O	O
3. ____________________<br>Intensity: □ very light □ light □ moderate □ vigorous □ very vigorous	_____	_____	O	O	O	O	O

70) **Did you regularly do relaxation activities (e.g. yoga, meditation) <u>during</u> the quarantine period?** Yes □ No □

**Relaxation activity**	**How often per week?**	**For how many minutes each?**	**Indepen-dently**	**In a club**	**In a gym and/or with a coach**	**Media-/online-based offer**	**Other**
1. ____________________	_____	_____	O	O	O	O	O
2. ____________________	_____	_____	O	O	O	O	O
3. ____________________	_____	_____	O	O	O	O	O

71) **On average, how many hours per week did you usually spend sitting <u>during</u> the quarantine period? (e.g. in the car, on the sofa, while watching TV, ...)**

**At work**		**In your private environment**	
Office work without screen work	☐ h	Computer/phone/tablet:	☐ h
Office work with screen work (e.g. computer):	☐ h	TV	☐ h
Other: ____________________	☐ h	Reading/writing	☐ h
Other: ____________________	☐ h	Hobbies (sewing, handicrafts, etc.):	☐ h
		Other: ____________________	☐ h

72) **What else did you do to relax during the quarantine period (e.g. by reading, watching television, playing games…)?**
You can select multiple answers.

- Reading
- Playing board games
- Watching TV
- Exercising

- o Playing music
- o Crafting
- o Gardening
- o Hobby: ____________________
- o Hobby: ____________________
- o Other: ____________________

Lifestyle during the quarantine period – others

	**Never**	**1x/month**	**2-3x/ month**	**2-3x/ week**	**4 and more times/ week**
How often do you typically drink alcohol outside the quarantine period?	O	O	O	O	O
Did this change during the quarantine period ?	☐ No<br>☐ Yes, insofar as ________________________________				

One alcoholic beverage corresponds to e.g. a small bottle of beer with 0,33l, a small glass of wine with 0,125l, a glass of sparkling wine or a double drink/schnapps	**1 – 2**	**3 – 4**	**5 – 6**	**7 – 9**	**10 or more**
If you drink alcohol in a day, how many alcoholic beverages do you typically drink outside of quarantine time?	O	O	O	O	O
Did it change during the quarantine period?	☐ No<br>☐ Yes, insofar as ________________				

One alcoholic beverage corresponds to e.g. a small bottle of beer with 0,33l, a small glass of wine with 0,125l, a glass of sparkling wine or a double drink/schnapps	**Never**	**Less than 1x/ month**	**1x/ month**	**1x/week**	**Daily or almost daily**
How many times did you drink more than 6 alcoholic beverages in one day during the quarantine period?	O	O	O	O	O
Did it change during the quarantine period?	☐ No<br>☐ Yes, insofar as ____________________				

73) Do you smoke? o No o Yes, since _______ years

74) If yes, how often do you smoke? o Daily o _______ days a week

75) How many cigarettes do you smoke? ____ per day

76) Did anything change in your smoking behaviour during the quarantine period?

- Yes, I smoke less
- Yes, I smoke more
- Yes, other: ____
- No

If you have any comments on individual questions or would you like to tell us anything else, you can do so here: ____

____

Thank you for your support!
